# Circulating Markers of Cardiovascular Health in Hypogonadism Before and After Testosterone Therapy: Molecular Aspects and Formulation Comparison

**DOI:** 10.3390/ijms27136035

**Published:** 2026-07-05

**Authors:** Sandro La Vignera, Rosita A. Condorelli

**Affiliations:** Department of Clinical and Experimental Medicine, University of Catania, 95124 Catania, Italy; rosita.condorelli@unict.it

**Keywords:** hypogonadism, testosterone replacement therapy, endothelial progenitor cells, endothelial microparticles, cardiovascular biomarkers, transdermal testosterone, intramuscular testosterone, androgen receptor signaling

## Abstract

Hypogonadism is increasingly recognized as an independent cardiovascular risk factor, with testosterone deficiency associated with endothelial dysfunction, increased thrombotic risk, and adverse cardiovascular outcomes. Circulating biomarkers provide valuable insights into the vascular health status of hypogonadal men and the cardiovascular effects of testosterone replacement therapy (TRT). This comprehensive review examines the molecular basis of testosterone action on the cardiovascular system and synthesizes evidence on circulating cardiovascular biomarkers in hypogonadism, including endothelial progenitor cells (EPCs), endothelial microparticles (EMPs), platelet markers, endothelial activators, adhesion molecules, and inflammatory/oxidative stress markers. We also compare the cardiovascular safety profiles of transdermal versus intramuscular testosterone formulations. Hypogonadal men exhibit reduced circulating EPCs, elevated EMPs, increased platelet reactivity, higher levels of endothelial activators (ICAM-1, VCAM-1, E-selectin, von Willebrand factor, endothelin-1, ADMA), and increased inflammatory markers (hsCRP, IL-6, TNF-α). TRT improves most of these biomarkers through androgen receptor (AR)-dependent and AR-independent mechanisms involving PI3K/Akt/eNOS signaling, VEGF upregulation, CXCL12/CXCR4 axis modulation, and NF-κB pathway suppression. Current evidence suggests that transdermal testosterone formulations may offer advantages regarding hematological safety and more stable testosterone exposure; however, definitive evidence demonstrating superior cardiovascular outcomes compared with intramuscular formulations remains limited. Circulating cardiovascular biomarkers are significantly altered in hypogonadism and improve with TRT. Available data suggest that transdermal testosterone formulations may offer a more favorable cardiovascular safety profile than intramuscular preparations, particularly with respect to erythrocytosis and pharmacokinetic stability, although head-to-head randomized trials with hard cardiovascular endpoints are still needed. Understanding the molecular mechanisms underlying these changes is essential for optimizing TRT in hypogonadal men with cardiovascular risk factors. The cardiovascular safety advantage of transdermal formulations is currently supported primarily by pharmacokinetic and hematological evidence; direct comparative evidence from randomized trials with hard cardiovascular endpoints remains unavailable.

## 1. Introduction

Hypogonadism, defined as testosterone deficiency with associated clinical symptoms, affects approximately 2–6% of men, with prevalence increasing substantially with age [1]. Beyond its well-recognized effects on sexual function, bone density, and muscle mass, testosterone deficiency has emerged as both a potential causal factor and a marker of poor cardiometabolic health, associated with increased cardiovascular risk [2,3]. Whether low testosterone acts as an independent causal risk factor or primarily reflects underlying metabolic dysfunction remains an area of active investigation. Epidemiological studies consistently demonstrate that low testosterone levels are associated with increased cardiovascular morbidity and mortality, including higher rates of myocardial infarction, stroke, and heart failure [4,5].

The mechanisms linking hypogonadism to cardiovascular disease are multifactorial and involve endothelial dysfunction, increased arterial stiffness, adverse lipid profiles, insulin resistance, increased visceral adiposity, chronic inflammation, and prothrombotic states [6,7]. Circulating biomarkers provide valuable windows into these pathophysiological processes, offering objective measures of vascular health that complement traditional cardiovascular risk assessment [8].

Endothelial progenitor cells (EPCs) are bone marrow-derived cells that contribute to vascular repair and neovascularization [9]. Reduced EPC numbers and impaired EPC function are associated with endothelial dysfunction and increased cardiovascular risk [9]. Conversely, endothelial microparticles (EMPs) are membrane vesicles released from activated or apoptotic endothelial cells that serve as biomarkers of endothelial damage and carry pro-inflammatory and pro-thrombotic cargo [10]. Platelet activation markers, endothelial adhesion molecules, and inflammatory cytokines further characterize the cardiovascular risk profile in hypogonadal men [11,12].

Testosterone replacement therapy (TRT) has been shown to improve several cardiovascular risk factors and biomarkers in hypogonadal men [13,14]. However, the cardiovascular safety of TRT remains a subject of ongoing debate, with concerns raised about potential adverse effects on thrombotic risk, particularly with certain formulations [15,16]. The pharmacokinetic profiles of different testosterone preparations—transdermal gels and patches versus intramuscular injections—differ substantially, with potential implications for cardiovascular safety [17,18].

This comprehensive review aims to: (1) elucidate the molecular basis of testosterone action on the cardiovascular system, with emphasis on androgen receptor (AR) signaling, genomic and non-genomic pathways, nitric oxide (NO) production, and inflammatory modulation; (2) systematically examine circulating cardiovascular biomarkers in hypogonadism, including EPCs, EMPs, platelet markers, endothelial activators, adhesion molecules, and inflammatory/oxidative stress markers; (3) evaluate the effects of TRT on these biomarkers and the underlying molecular mechanisms; (4) compare the cardiovascular safety profiles of transdermal versus intramuscular testosterone formulations, with emphasis on pharmacokinetic differences and their clinical implications.

## 2. Molecular Basis of Testosterone Action on the Cardiovascular System

### 2.1. Androgen Receptor Signaling: Genomic and Non-Genomic Pathways

Testosterone exerts its cardiovascular effects through both genomic (classical) and non-genomic (rapid) mechanisms [19,20]. The genomic pathway involves testosterone binding to intracellular androgen receptors (AR) or conversion to dihydrotestosterone (DHT) by 5α-reductase, followed by AR dimerization, nuclear translocation, and transcriptional regulation of target genes [21]. Functional ARs are expressed in cardiomyocytes, vascular endothelial cells, and vascular smooth muscle cells (VSMCs), enabling direct cardiovascular effects [22,23].

Non-genomic actions of testosterone occur within seconds to minutes, too rapidly to involve gene transcription [20]. These rapid effects are mediated by membrane-associated ARs or G-protein-coupled receptors that activate intracellular signaling cascades, including protein kinase A (PKA), protein kinase C (PKC), mitogen-activated protein kinases (MAPKs), and phosphatidylinositol 3-kinase (PI3K)/Akt pathways [24,25]. Non-genomic mechanisms also involve direct modulation of ion channels, including L-type voltage-operated calcium channels and ATP-sensitive potassium channels, leading to rapid vasodilation [22,26].

### 2.2. Nitric Oxide Production and Endothelial Function

A central mechanism by which testosterone promotes cardiovascular health is through enhancement of endothelial nitric oxide (NO) production [23,27]. Testosterone rapidly activates endothelial nitric oxide synthase (eNOS) via AR-dependent signaling involving Src kinase and the PI3K/Akt pathway [23,24]. Yu et al. demonstrated that testosterone at physiological concentrations (1–100 nM) rapidly induced NO production and eNOS phosphorylation at Ser1177 in human aortic endothelial cells through an AR-dependent, PI3K/Akt-mediated mechanism [23]. This effect was abolished by AR antagonists or AR siRNA, confirming AR dependence, and was independent of aromatization to estradiol [23].

The Src kinase-PI3K/Akt-eNOS signaling cascade represents a critical non-genomic pathway linking testosterone to endothelial function [24]. Src kinase mediates AR-dependent rapid phosphorylation of Akt, which in turn phosphorylates eNOS at Ser1177, increasing its enzymatic activity and NO production [24]. Enhanced NO bioavailability promotes vasodilation, inhibits platelet aggregation, reduces leukocyte adhesion to the endothelium, and prevents VSMC proliferation—all atheroprotective effects [20].

Testosterone also regulates eNOS expression at the transcriptional level through genomic mechanisms [28]. In testosterone-deficient states, eNOS expression and activity are reduced, leading to decreased NO bioavailability and endothelial dysfunction [27]. Testosterone replacement therapy restores eNOS expression and phosphorylation, improving endothelial function [29].

### 2.3. Vascular Smooth Muscle Relaxation and Ion Channel Modulation

Testosterone induces vascular smooth muscle relaxation through multiple mechanisms [20]. Non-genomic actions include direct inhibition of L-type voltage-operated calcium channels, reducing intracellular calcium influx and promoting vasodilation [20,22]. Testosterone also activates ATP-sensitive potassium channels (KATP) in VSMCs, leading to membrane hyperpolarization and reduced calcium entry [30]. These rapid vasodilatory effects occur independently of the endothelium and contribute to testosterone’s acute anti-ischemic properties [31].

Testosterone influences vascular reactivity by regulating VSMC calcium homeostasis, Rho kinase activation, and mitogen-activated protein kinase phosphorylation [32,33]. In hypogonadal states, upregulation of the RhoA/Rho-kinase (ROCK) signaling pathway increases vascular contractility and contributes to endothelial dysfunction [3]. Testosterone replacement reverses this upregulation, reducing vascular tone and improving endothelial function [3].

### 2.4. Oxidative Stress and Reactive Oxygen Species

The relationship between testosterone and oxidative stress is complex and dose-dependent [34,35]. At physiological concentrations, testosterone exhibits antioxidant properties, suppressing oxidative stress and enhancing antioxidant enzyme activities [35]. Zhang et al. demonstrated that testosterone deficiency induces oxidative stress in cardiomyocytes, decreasing superoxide dismutase (SOD) and glutathione peroxidase (GSH-Px) activities and increasing malondialdehyde (MDA) levels and mitochondrial DNA mutations [35]. Physiological testosterone replacement reversed these changes via an AR-independent pathway [35].

However, supraphysiological testosterone levels can induce oxidative stress and vascular dysfunction [26,36]. Alves et al. showed that supraphysiological testosterone induces vascular dysfunction via mitochondrial reactive oxygen species (mROS) generation and activation of the NLRP3 inflammasome, leading to increased caspase-1 and IL-1β expression [26]. Testosterone can stimulate NADPH oxidase activity in VSMCs, increasing superoxide anion production through AR-dependent mechanisms [36,37]. Costa et al. demonstrated that testosterone promotes nuclear factor E2-related factor 2 (Nrf2) downregulation, compromising antioxidant defenses and exacerbating oxidative stress in the context of high-fat diet-induced vascular dysfunction [36].

These findings underscore the importance of maintaining physiological testosterone levels and avoiding supraphysiological peaks that may promote oxidative stress and vascular damage [35].

### 2.5. Inflammatory Pathways and NF-κB Signaling

Testosterone exerts anti-inflammatory effects on the vascular endothelium through multiple mechanisms [6,19]. Testosterone inhibits nuclear factor-κB (NF-κB) activation, a master regulator of inflammatory gene expression [19]. By suppressing NF-κB signaling, testosterone reduces the expression of pro-inflammatory cytokines (tumor necrosis factor-α [TNF-α], interleukin-1β [IL-1β], interleukin-6 [IL-6]) and endothelial adhesion molecules (vascular cell adhesion molecule-1 [VCAM-1], intercellular adhesion molecule-1 [ICAM-1], E-selectin) [7,19].

Conversely, testosterone stimulates the production of the anti-inflammatory cytokine interleukin-10 (IL-10), further contributing to its anti-inflammatory profile [3]. Dihydrotestosterone (DHT) has been shown to decrease TNF-α and lipopolysaccharide (LPS)-induced inflammatory responses in human endothelial cells [38]. In hypogonadal states, elevated levels of pro-inflammatory cytokines contribute to endothelial dysfunction, increased atherosclerotic plaque formation, and heightened cardiovascular risk [3].

### 2.6. Endothelial Progenitor Cell Mobilization and Angiogenesis

Testosterone promotes endothelial progenitor cell (EPC) mobilization from bone marrow and enhances EPC proliferation, colony formation, and migration through AR-mediated pathways [39,40]. The CXCL12/CXCR4 chemokine axis plays a critical role in EPC mobilization, and testosterone upregulates this signaling pathway [27]. Vascular endothelial growth factor (VEGF) is another key mediator of EPC function and angiogenesis that is positively regulated by testosterone [41,42].

Yoshida et al. demonstrated that AR activation leads to rapid NO production via eNOS phosphorylation, involving Src kinase and the PI3K/Akt cascade, and that VEGF-stimulated Akt and eNOS phosphorylation are blunted by AR deficiency [43]. Androgens increase EPC mobilization from bone marrow and promote proliferation, colony formation, and migration via AR-mediated pathways [44]. AR deficiency impairs eNOS expression and phosphorylation, leading to reduced NO bioavailability and accelerated vascular remodeling [45].

Testosterone also ameliorates endothelial senescence, preserving vascular regenerative capacity [46]. In castrated animal models, circulating EPC numbers decrease significantly, and testosterone replacement restores EPC levels [40,47]. These effects contribute to vascular repair, neovascularization, and maintenance of endothelial integrity [48].

## 3. Circulating Biomarkers of Cardiovascular Health in Hypogonadism

### 3.1. Endothelial Progenitor Cells (EPCs)

#### 3.1.1. Definition and Subpopulations

Endothelial progenitor cells (EPCs) are bone marrow-derived cells that circulate in peripheral blood and contribute to endothelial repair, neovascularization, and maintenance of vascular homeostasis [49]. EPCs are typically identified by co-expression of stem cell markers (CD34, CD133) and endothelial markers (kinase insert domain receptor [KDR]/VEGFR-2, CD144/VE-cadherin) [8]. Common EPC phenotypes include CD34+/KDR+, CD133+/KDR+, and CD45neg/CD34+/CD144+ cells [8]. It should be noted that EPC characterization remains methodologically controversial: different studies employ different surface marker combinations (e.g., CD34+/KDR+, CD133+/KDR+, CD45neg/CD34+/CD144+), which results in substantial variability in reported cell counts and functional properties across the literature. This heterogeneity should be considered when comparing findings from different studies.

EPCs can be further classified into early “monocytic” EPCs and late “endothelial colony-forming” EPCs based on their culture characteristics and functional properties [40]. Early EPCs appear within days in culture, exhibit limited proliferative capacity, and primarily exert paracrine effects by secreting angiogenic factors [40]. Late EPCs emerge after 2–3 weeks, demonstrate high proliferative potential, and directly incorporate into vascular structures [40].

#### 3.1.2. Role in Vascular Repair and Cardiovascular Risk

EPCs play a critical role in endothelial repair by homing to sites of vascular injury, differentiating into mature endothelial cells, and promoting re-endothelialization [50]. Reduced circulating EPC numbers and impaired EPC function are associated with endothelial dysfunction, increased atherosclerotic burden, and adverse cardiovascular outcomes [9,51]. Low EPC levels predict future cardiovascular events independently of traditional risk factors [9].

#### 3.1.3. EPC Levels in Hypogonadal Men

Multiple studies have demonstrated reduced circulating EPC numbers in hypogonadal men compared to eugonadal controls [13,51,52]. Foresta et al. reported significantly reduced numbers of circulating progenitor cells (PCs) and EPCs in young hypogonadal men, with this reduction attributed to a possible direct effect on bone marrow [52]. Mambro et al. found that Klinefelter syndrome (KS) patients without cardiovascular risk factors had significantly lower EPC levels than controls without cardiovascular risk factors, suggesting that reduced EPCs might be an independent cardiovascular risk factor in KS [51].

La Vignera et al. identified a novel immunophenotype of blood EPCs (CD45neg/CD34+/CD144+) and endothelial microparticles in patients with arterial erectile dysfunction and late-onset hypogonadism [8]. Both EPCs and EMPs were significantly higher in patients with erectile dysfunction (ED) and late-onset hypogonadism (LOH) compared with patients with ED alone or controls, and higher in ED alone patients than controls, suggesting LOH is an additional vascular risk factor [8].

Milardi et al. demonstrated that circulating endothelial cell (CEC) count was significantly increased in patients with hypogonadism compared to controls, with an inverse exponential correlation between testosterone levels and CEC count [12]. Direct linear correlations were found between waist circumference and CECs, and between BMI and CECs, with testosterone identified as the significant independent determinant of CECs [12].

#### 3.1.4. Molecular Mechanisms Linking Low Testosterone to Reduced EPC Mobilization

The mechanisms by which testosterone deficiency reduces EPC numbers involve multiple signaling pathways [27]. The CXCL12/CXCR4 chemokine axis is critical for EPC mobilization from bone marrow, and testosterone upregulates this pathway [27]. Testosterone also enhances eNOS activity and NO production, which promote EPC mobilization and function [27]. VEGF, a key regulator of EPC proliferation and angiogenesis, is positively regulated by testosterone [53].

Hotta et al. reviewed that testosterone deficiency affects EPC function and number, and that testosterone replacement therapy improved EPC function [27]. Testosterone deficiency might cause endothelial dysfunction by decreasing nitric oxide (NO) levels through regulating the expression and activity of NO synthase and increasing asymmetric dimethylarginine (ADMA) expression [27]. Testosterone also regulates phosphodiesterase type 5 (PDE-5) expression [27].

Fadini et al. found that testosterone and dihydrotestosterone had no effect on late EPCs from healthy human adult males but positively modulated early “monocytic” EPCs [40]. Castration in rats decreased circulating EPCs, but testosterone/dihydrotestosterone replacement failed to restore them [40]. In men, circulating EPC levels were more associated with estradiol than testosterone, suggesting that androgens may exert no direct effects on EPC biology or that effects are mediated through aromatization [40].

### 3.2. Endothelial Microparticles (EMPs)

#### 3.2.1. Definition and Surface Markers

Endothelial microparticles (EMPs) are small membrane vesicles (0.1–1.0 μm) released from endothelial cells during activation, apoptosis, or injury [10]. EMPs express endothelial surface markers, including CD31 (platelet endothelial cell adhesion molecule-1), CD51 (integrin αV), CD62E (E-selectin), and CD144 (VE-cadherin) [8]. EMPs also express phosphatidylserine on their surface, which can be detected by annexin V binding [8].

#### 3.2.2. Role as Biomarkers of Endothelial Damage

Elevated circulating EMP levels reflect endothelial activation, dysfunction, and damage [10]. EMPs serve as biomarkers of endothelial injury in various cardiovascular conditions, including atherosclerosis, hypertension, diabetes, and acute coronary syndromes [10]. EMP levels correlate with endothelial dysfunction assessed by flow-mediated dilation and predict cardiovascular events [10].

#### 3.2.3. Pro-Thrombotic and Pro-Inflammatory Cargo

EMPs carry bioactive molecules, including adhesion molecules, cytokines, chemokines, and tissue factor, which can promote inflammation, coagulation, and vascular dysfunction [10]. EMPs can activate platelets, induce endothelial cell apoptosis, impair endothelial function, and promote atherosclerotic plaque formation [10]. The pro-thrombotic properties of EMPs are mediated by surface expression of tissue factor and phosphatidylserine, which provide a catalytic surface for coagulation reactions [10].

#### 3.2.4. Elevated EMPs in Hypogonadism

La Vignera et al. reported that endothelial microparticles (CD45neg/CD144+/annexin V+) were significantly higher in patients with erectile dysfunction and late-onset hypogonadism compared with patients with ED alone or controls [8]. Omar et al. found that mean EMP levels were 0.15 ± 0.029 in vasculogenic ED with LOH, 0.056 ± 0.013 in vasculogenic ED without LOH, and 0.014 ± 0.002 in controls, demonstrating a significant association between LOH and higher expression of EMPs in patients with vasculogenic ED [54].

These findings suggest that hypogonadism is associated with increased endothelial damage and activation, as reflected by elevated EMP levels [8]. The combination of reduced EPCs (impaired vascular repair capacity) and elevated EMPs (increased endothelial damage) creates a particularly adverse cardiovascular risk profile in hypogonadal men [8].

### 3.3. Platelet Markers

#### 3.3.1. Platelet Activation Markers

Platelet activation plays a central role in thrombosis and cardiovascular events [11]. Key markers of platelet activation include P-selectin (CD62P), glycoprotein IIb/IIIa (GPIIb/IIIa), and thromboxane A2 (TXA2) [11]. P-selectin is stored in α-granules and translocated to the platelet surface upon activation, where it mediates platelet-leukocyte interactions [11]. GPIIb/IIIa is the fibrinogen receptor that mediates platelet aggregation [11]. TXA2 is a potent platelet agonist and vasoconstrictor synthesized by activated platelets [11].

#### 3.3.2. Platelet-Derived Microparticles (PDMPs)

Platelet-derived microparticles (PDMPs) are membrane vesicles released from activated platelets that express platelet surface markers (CD41, CD42b, CD62P) and carry pro-coagulant phospholipids [11]. PDMPs amplify coagulation, promote thrombin generation, and contribute to thrombotic risk [11].

#### 3.3.3. Platelet Aggregation in Testosterone-Deficient Men

Evidence regarding platelet function in hypogonadism is mixed. Minno et al. reported increased platelet reactivity in Klinefelter syndrome (KS) men on testosterone replacement therapy, with maximal platelet aggregation (Max-A%) higher in KS than controls (65.61% vs. 46.30% with 0.2 mM arachidonic acid; 96.43% vs. 81.04% with 0.4 mM arachidonic acid) [11]. Oxidative stress markers 8-iso-prostaglandin F2α (446.54 pg/mg creatinine vs. 230.00 pg/mg creatinine) and 11-dehydro-thromboxane-B2 (1278.36 pg/mg creatinine vs. 595.08 pg/mg creatinine) were also higher in KS, suggesting these contribute to increased thrombotic risk [11].

However, Chang et al. found that platelet aggregation in Klinefelter syndrome is not aggravated by testosterone replacement therapy in a longitudinal follow-up study [28]. Faraday et al. reported increased platelet aggregation activity and thromboxane A2 receptor density with testosterone therapy, and increased P2Y12 gene expression in culture [55]. Testosterone may promote arterial thrombosis via increased P2Y12 gene expression on platelets [56].

#### 3.3.4. Molecular Pathways

The molecular mechanisms linking testosterone to platelet function involve multiple pathways [57]. Testosterone can stimulate P2Y12 gene expression, enhancing platelet responsiveness to ADP [58]. Oxidative stress induced by testosterone may increase thromboxane synthesis and platelet activation [11]. However, testosterone also stimulates nitric oxide production, which inhibits platelet aggregation, suggesting complex and potentially opposing effects [20].

### 3.4. Endothelial Activators and Adhesion Molecules

#### 3.4.1. ICAM-1, VCAM-1, and E-Selectin

Intercellular adhesion molecule-1 (ICAM-1), vascular cell adhesion molecule-1 (VCAM-1), and E-selectin are endothelial adhesion molecules that mediate leukocyte recruitment to the vascular wall, a critical early step in atherogenesis [19]. These molecules are upregulated by inflammatory cytokines and oxidative stress through NF-κB-dependent mechanisms [19].

Fahed et al. reported that VCAM levels inversely correlated with testosterone levels in male coronary artery disease (CAD) patients, although testosterone replacement did not affect serum VCAM levels in hypogonadal men [3]. Kelly et al. noted that testosterone inhibits NF-κB activation, reducing inflammatory cytokines (TNF-α, IL-1β, IL-6) and adhesion molecules (VCAM-1, ICAM-1) [19].

#### 3.4.2. Von Willebrand Factor (vWF)

Von Willebrand factor (vWF) is a large multimeric glycoprotein synthesized by endothelial cells and megakaryocytes that mediates platelet adhesion to damaged endothelium and serves as a carrier protein for factor VIII [59]. Elevated vWF levels reflect endothelial activation and dysfunction and predict cardiovascular events [60]. Limited data exist on vWF levels in hypogonadism, but endothelial dysfunction in hypogonadal men would be expected to increase vWF release [61].

#### 3.4.3. Endothelin-1 (ET-1)

Endothelin-1 (ET-1) is a potent vasoconstrictor peptide produced by endothelial cells that contributes to vascular tone regulation, vascular remodeling, and atherosclerosis [3]. Elevated ET-1 levels are associated with endothelial dysfunction, hypertension, and cardiovascular disease [3]. Fahed et al. reported that ET-1 was elevated in hypogonadal men and reduced with testosterone treatment [3].

#### 3.4.4. Asymmetric Dimethylarginine (ADMA)

Asymmetric dimethylarginine (ADMA) is an endogenous inhibitor of nitric oxide synthase that reduces NO production and impairs endothelial function [27]. Elevated ADMA levels are associated with endothelial dysfunction, atherosclerosis, and cardiovascular events [27]. Fahed et al. reported that ADMA was increased in the hypogonadal state and reversed by testosterone administration [3]. Hotta et al. noted that testosterone deficiency increases ADMA expression, contributing to decreased NO production and endothelial dysfunction [27].

### 3.5. Inflammatory and Oxidative Markers

#### 3.5.1. High-Sensitivity C-Reactive Protein (hsCRP)

High-sensitivity C-reactive protein (hsCRP) is a sensitive marker of systemic inflammation and an independent predictor of cardiovascular events [62]. Elevated hsCRP levels are associated with increased risk of myocardial infarction, stroke, and cardiovascular death [62]. Fahed et al. reported an inverse correlation between testosterone levels and CRP, with androgen deprivation therapy (ADT) patients showing increased CRP [3]. However, testosterone replacement was not consistently shown to affect CRP levels [3]. It is important to note that findings across studies are not entirely consistent: while some studies report reductions in hsCRP with TRT, others, including the large Testosterone Trials, found no significant change in CRP or IL-6 [29]. This heterogeneity may reflect differences in patient populations, testosterone formulations, doses, and duration of treatment. Importantly, improvements in inflammatory biomarkers do not necessarily translate into reductions in major cardiovascular events, and this distinction should be borne in mind when interpreting these findings.

Ezeamii et al. reported that testosterone replacement therapy decreased pro-inflammatory cytokines (IL-6, TNF-α) and enhanced endothelial-dependent vasodilation [14]. Mohler et al. found that testosterone treatment in the Testosterone Trials significantly decreased total cholesterol by 6.1 mg/dL, HDL by 2.0 mg/dL, and fasting insulin by 1.7 μIU/mL, with HOMA-IR decreasing by 0.6, but C-reactive protein and interleukin-6 showed no significant change [29].

#### 3.5.2. Interleukin-6 (IL-6) and Tumor Necrosis Factor-α (TNF-α)

Interleukin-6 (IL-6) and tumor necrosis factor-α (TNF-α) are pro-inflammatory cytokines that contribute to endothelial dysfunction, atherosclerosis, and cardiovascular disease [63]. Elevated levels of these cytokines are associated with increased cardiovascular risk [64]. Fahed et al. reported that testosterone replacement decreased levels of TNF-α, IL-6, and IL-1β in vitro and increased IL-10 [3]. Faraday et al. noted that elevated IL-1β was found in hypogonadal men, and testosterone therapy reduced TNF-α and IL-1β in hypogonadal men [65]. However, the evidence for TRT-mediated reductions in IL-6 and TNF-α is not uniform across studies, and the clinical significance of biomarker improvements in terms of hard cardiovascular outcomes remains uncertain.

Vatti et al. reported that TNF-α and IL-6 levels declined with DHEA supplementation and testosterone undecanoate (TU) therapy, and that hsCRP levels significantly decreased with TU therapy and were higher in low testosterone groups [66].

#### 3.5.3. Oxidized LDL and Malondialdehyde

Oxidized low-density lipoprotein (oxLDL) is a key mediator of atherosclerosis that promotes endothelial dysfunction, foam cell formation, and inflammatory responses [67]. Malondialdehyde (MDA) is a product of lipid peroxidation that serves as a biomarker of oxidative stress [68]. Minno et al. reported higher levels of 8-iso-prostaglandin F2α (a marker of oxidative stress) in Klinefelter syndrome patients [11].

#### 3.5.4. Superoxide Dismutase (SOD)

Superoxide dismutase (SOD) is a key antioxidant enzyme that catalyzes the dismutation of superoxide radicals to hydrogen peroxide and oxygen [69]. Reduced SOD activity reflects impaired antioxidant defenses and increased oxidative stress [70]. Zhang et al. demonstrated that testosterone deficiency decreases SOD and glutathione peroxidase (GSH-Px) enzyme activities, and that physiological testosterone therapy reverses these changes, increasing SOD and GSH-Px activities [35].

## 4. Effect of Testosterone Replacement Therapy (TRT) on Circulating Cardiovascular Biomarkers

### 4.1. Changes in EPCs After TRT

Multiple studies have demonstrated that testosterone replacement therapy increases circulating EPC numbers in hypogonadal men [13,22,52]. Liao et al. reported that before TRT, there was no significant association between serum total testosterone and EPC number, but after TRT with transdermal testosterone gel (Androgel; 1% testosterone at 5 g/day), the mean circulating EPC number significantly increased from 9.5 ± 6.2 at baseline to 16.6 ± 11.1 (*p* = 0.027) after 3 months, 20.3 ± 15.3 (*p* = 0.006) after 6 months, and 27.2 ± 15.5 (*p* = 0.017) after 12 months [13].

Foresta et al. reported that testosterone treatment for 6 months was able to induce an increase in circulating PCs and EPCs in young hypogonadal men, with this increase attributed to a possible direct effect on the bone marrow [52]. Francomano et al. noted that transdermal testosterone administration was able to increase the number of circulating EPCs in a previous study [22].

However, not all studies have shown consistent effects. Mambro et al. found that six months of androgen therapy in 22 hypogonadal Klinefelter syndrome patients did not modify EPC number, suggesting that factors related to the supernumerary X chromosome might reduce EPC numbers independently of testosterone levels [51]. Ru et al. reported that testosterone level does not significantly correlate with circulating endothelial progenitor cells in Klinefelter’s syndrome patients [71].

The mechanisms by which TRT increases EPC numbers involve upregulation of the CXCL12/CXCR4 axis, enhanced eNOS activity and NO production, and increased VEGF expression [27,72]. These pathways promote EPC mobilization from bone marrow, proliferation, and homing to sites of vascular injury [65].

### 4.2. Changes in EMPs After TRT

Limited data exist on the effects of TRT on endothelial microparticle levels. Given that EMPs reflect endothelial damage and activation, and that TRT improves endothelial function, it would be expected that TRT would reduce EMP levels [10]. However, specific studies quantifying EMP changes with TRT are needed to confirm this hypothesis [10]. This represents a promising and underexplored direction for future investigation. Furthermore, EMP quantification lacks standardization: pre-analytical variables (e.g., centrifugation speed, time to processing, anticoagulant used) and analytical factors (e.g., flow cytometry gating strategy, choice of surface markers such as CD31+/CD41−, CD51+, or CD144+) significantly affect reported concentrations, making cross-study comparisons challenging. Consensus guidelines for EMP measurement are needed before EMP levels can be reliably used as clinical biomarkers in the context of TRT.

### 4.3. Changes in Platelet Markers After TRT

The effects of TRT on platelet function remain controversial, and it is important to distinguish between physiological testosterone replacement and supraphysiological androgen exposure, as the two may have divergent effects. Some studies suggest that testosterone at supraphysiological concentrations may increase platelet reactivity and thrombotic risk, while evidence from conventional TRT aiming at physiological restoration is less consistent [11,28]. Minno et al. found increased platelet reactivity in Klinefelter syndrome men on testosterone replacement therapy [11]; however, this population has unique genetic characteristics that may not be representative of conventional hypogonadal men. Chang et al. demonstrated that platelet aggregation in Klinefelter syndrome is not aggravated by testosterone replacement therapy in a longitudinal follow-up study [28]. Much of the evidence linking testosterone to increased platelet activation derives from studies using supraphysiological doses or specific clinical settings (e.g., anabolic steroid abuse, Klinefelter syndrome), and may not be directly applicable to conventional TRT at physiological replacement doses.

Faraday et al. reported that testosterone therapy was associated with increased platelet aggregation activity and thromboxane A2 receptor density, and increased P2Y12 gene expression [73]. However, a randomized, double-blinded, placebo-controlled study using a transdermal testosterone patch showed no changes in plasma levels of fibrinogen, tissue plasminogen activator (tPA), or plasminogen activator inhibitor-1 (PAI-1) over three months in men with stable coronary heart disease [74]. Testosterone administered by injection over 52 weeks was associated with an initial fall in PAI-1, protein C, and protein S [60].

The balance between pro-thrombotic effects (increased platelet activation, P2Y12 expression) and anti-thrombotic effects (increased NO production, reduced PAI-1) may depend on testosterone dose, formulation, and individual patient characteristics [57].

### 4.4. Changes in Endothelial Activators and Adhesion Molecules After TRT

Testosterone replacement therapy has been shown to improve endothelial function and reduce markers of endothelial activation [22,73]. Shoskes et al. reported that mean reactive hyperemia index (RHI) improved from 1.70 to 2.14 (*p* = 0.01) and mean augmentation index (AI) improved from 2.9% to -1.75% (*p* = 0.01) after testosterone replacement therapy [75]. Endothelial function either remained unchanged or improved following TRT, with no man experiencing worsened AI [22].

Francomano et al. found that after transdermal testosterone gel administration, RHI significantly improved at 4 h, and AI improved at 4 h and 96 h [22]. These improvements in endothelial function suggest reduced endothelial activation and potentially lower levels of adhesion molecules and endothelial activators [22].

Fahed et al. reported that endothelin-1 (ET-1) was elevated in hypogonadal men and reduced with testosterone treatment [3]. Asymmetric dimethylarginine (ADMA) was increased in the hypogonadal state and reversed by testosterone administration [3]. These findings indicate that TRT improves endothelial function by reducing vasoconstrictor and eNOS inhibitor levels [3].

### 4.5. Changes in Inflammatory and Oxidative Markers After TRT

Testosterone replacement therapy has been shown to reduce inflammatory markers in several studies [3,14]. Fahed et al. reported that testosterone replacement decreased levels of TNF-α, IL-6, and IL-1β in vitro and increased IL-10 [3]. Faraday et al. noted that testosterone therapy reduced TNF-α and IL-1β in hypogonadal men [72]. Ezeamii et al. reported that TRT decreased pro-inflammatory cytokines (IL-6, TNF-α) [14].

Vatti et al. found that TNF-α and IL-6 levels declined with DHEA supplementation and testosterone undecanoate therapy, and that hsCRP levels significantly decreased with TU therapy [66]. However, Mohler et al. reported that in the Testosterone Trials, C-reactive protein and interleukin-6 showed no significant change with testosterone treatment [29].

Regarding oxidative stress markers, Zhang et al. demonstrated that physiological testosterone therapy increases SOD and GSH-Px activities and decreases MDA levels and mitochondrial DNA mutations [35]. These findings suggest that TRT at physiological doses improves antioxidant defenses and reduces oxidative stress [35].

#### Conflicting Evidence and the Distinction Between Biomarker Changes and Cardiovascular Outcomes

While several studies report improvements in inflammatory and oxidative biomarkers with TRT, the literature remains heterogeneous regarding actual cardiovascular outcomes. The large TRAVERSE trial (*n* = 5246), a randomized double-blind placebo-controlled study, demonstrated that TRT with transdermal testosterone gel was non-inferior to placebo for major adverse cardiovascular events (MACE), providing important reassurance regarding cardiovascular safety in men at elevated cardiovascular risk [76]. However, the Testosterone Trials substudy (Shaikh et al.) found that testosterone treatment was associated with greater progression of non-calcified coronary plaque, raising concerns about potential atherogenic effects in some populations [29]. These contrasting findings underscore that improvements in surrogate biomarkers (hsCRP, IL-6, TNF-α, endothelial function markers) do not necessarily translate into reductions in major cardiovascular events. Mechanistic evidence—such as activation of PI3K/Akt/eNOS signaling or reductions in inflammatory cytokines—should not be interpreted as direct evidence of reduced cardiovascular events unless supported by outcome data. A dedicated analysis of conflicting evidence, including observational studies, RCTs, and meta-analyses, is essential for a balanced interpretation of TRT’s cardiovascular effects. This distinction is fundamental: improvements in surrogate biomarkers, however consistent, cannot substitute for evidence from randomized trials with pre-specified hard cardiovascular endpoints (myocardial infarction, stroke, cardiovascular death) when drawing conclusions about clinical benefit.

## 5. Transdermal vs. Intramuscular Testosterone: Formulation Comparison and Cardiovascular Safety

### 5.1. Pharmacokinetics: Steady-State vs. Supraphysiological Peaks

The pharmacokinetic profiles of testosterone formulations differ substantially and have important implications for cardiovascular safety [17,18]. Transdermal testosterone formulations (gels, patches) provide continuous, steady-state testosterone levels that mimic physiological circadian variations [17]. In contrast, intramuscular (IM) testosterone injections produce supraphysiological peaks shortly after administration, followed by a gradual decline to hypogonadal or low-normal levels before the next injection [17,77].

Dobs et al. compared a permeation-enhanced testosterone transdermal system (Androderm) with bi-weekly injections of testosterone enanthate in hypogonadal men [17]. The transdermal system produced physiological sex hormone levels with circadian variations, while IM testosterone enanthate produced supraphysiological levels of testosterone, bioavailable testosterone, and estradiol for several days post-injection [17]. IM treatment was associated with significantly more abnormal hematocrit elevations (43.8% of patients) compared with the transdermal system (15.4% of patients) [17].

Bassil et al. noted that intramuscular injections often cause supraphysiological peaks and hypogonadal troughs, leading to fluctuating testosterone levels, while transdermal systems provide continuous, more uniform physiological levels [78]. Supraphysiological testosterone levels can increase LDL cholesterol and decrease HDL cholesterol, potentially increasing cardiovascular risk [76].

Peña et al. reported that transdermal gel provides stable serum levels mimicking circadian rhythm, while injectable testosterone undecanoate shows stable absorption without supra- or infra-physiological variations, but intramuscular cypionate, enanthate, and propionate cause initial supraphysiological levels followed by subnormal drops [77]. Testosterone replacement therapy can increase hematocrit and erythrocytosis risk, and genetic predisposition to high endogenous testosterone is associated with heart failure and thromboembolism [79].

### 5.2. Impact of Pharmacokinetic Profile on Cardiovascular Biomarkers

The supraphysiological testosterone peaks associated with IM injections may have adverse effects on cardiovascular biomarkers and thrombotic risk [11,26]. Supraphysiological testosterone levels can induce oxidative stress, activate the NLRP3 inflammasome, increase platelet reactivity, and promote erythrocytosis [18,26]. Alves et al. demonstrated that supraphysiological testosterone induces vascular dysfunction via mitochondrial ROS generation and NLRP3 inflammasome activation [26].

In contrast, the steady-state physiological testosterone levels achieved with transdermal formulations are less likely to induce these adverse effects [17]. Physiological testosterone levels enhance NO production, reduce inflammation, and improve endothelial function without promoting excessive oxidative stress or erythrocytosis [23].

### 5.3. Evidence for Greater Cardiovascular Safety of Transdermal Formulations

Several studies have suggested that transdermal testosterone formulations may offer a more favorable cardiovascular safety profile compared to intramuscular injections, primarily with respect to erythrocytosis rates and pharmacokinetic variability [15,77]. However, it should be noted that current evidence is stronger for differences in hematological outcomes and pharmacokinetic stability than for differences in major cardiovascular endpoints, and robust head-to-head randomized trials with hard cardiovascular outcomes directly comparing the two routes of administration are still lacking. Anawalt et al. noted that findings suggest transdermal testosterone gels may be associated with more favorable cardiovascular outcomes compared to injectable testosterone esters, including lower rates of cardiovascular events, all-cause hospitalizations, and all-cause mortality [77], though these observations derive largely from observational and indirect comparisons. It is therefore important to emphasize that any apparent cardiovascular advantage of transdermal formulations currently rests primarily on pharmacokinetic and hematological data rather than on direct evidence from randomized trials powered for hard cardiovascular endpoints (Table 1).

Khera et al. reported that Layton et al. compared the cardiovascular safety of testosterone injections, patches, and gels, finding an increased risk in new testosterone injection initiators compared with gels [15]. Albert et al. found in a systematic review that intramuscular testosterone appeared neutral for cardiovascular events (RR = 0.96) compared with oral (RR = 2.28) and transdermal testosterone (RR = 2.80), and that intramuscular testosterone had the least effect of lowering HDL and non-HDL cholesterol [30]. However, this finding is inconsistent with other studies and may reflect study heterogeneity [30].

Shores et al. reported that in adjusted Cox regression analyses, current use of transdermal testosterone was not associated with risk for composite cardiovascular outcomes in those without prevalent cardiovascular disease (HR, 0.89; 95% CI, 0.76–1.05) and was associated with lower risk in those with prevalent cardiovascular disease (HR, 0.80; 95% CI, 0.70–0.91) [31]. Intramuscular testosterone showed no association with risk in either group [31].

Baillargeon et al. found no increased risk of venous thromboembolism (VTE) for specific routes of administration: topical (aOR, 0.80; 95% CI, 0.61–1.41), transdermal (aOR, 0.91; 95% CI, 0.38–2.16), and intramuscular (aOR, 1.15; 95% CI, 0.80–1.64) in a case–control study [44].

### 5.4. Hematocrit, Erythrocytosis, and Thrombotic Risk with IM Testosterone

Erythrocytosis (elevated hematocrit) is a well-recognized adverse effect of testosterone replacement therapy, particularly with intramuscular formulations [17,18]. Testosterone stimulates erythropoiesis through multiple mechanisms: (1) increased erythropoietin (EPO) production by the kidneys, mediated by androgen receptor activation in renal peritubular cells; (2) suppression of hepcidin, a key regulator of iron availability, leading to enhanced iron absorption and utilization for red blood cell synthesis; (3) direct stimulation of erythroid progenitor cells in the bone marrow, promoting their proliferation and differentiation; and (4) potential effects on hypoxia-inducible factor (HIF) pathways [32]. Elevated hematocrit increases blood viscosity, impairs microvascular blood flow, and increases thrombotic risk through enhanced platelet aggregation and reduced fibrinolysis [18]. The risk of erythrocytosis is formulation-dependent: intramuscular testosterone preparations, particularly short-acting esters (enanthate, cypionate) that produce supraphysiological peaks, carry the highest erythrocytosis risk. Long-acting intramuscular testosterone undecanoate carries intermediate risk, while transdermal formulations carry the lowest risk due to their stable, physiological testosterone exposure [17,18]. Regarding monitoring and management: hematocrit should be measured at baseline, at 3–6 months after TRT initiation, and then annually. TRT dose reduction or temporary discontinuation is recommended if hematocrit exceeds 54%. Therapeutic phlebotomy may be considered in selected cases. Switching from intramuscular to transdermal formulations is advisable in patients who develop erythrocytosis [80]. The clinical implications of TRT-induced erythrocytosis for venous thromboembolism risk remain an area of active investigation, and patients with pre-existing thrombophilia or prior thromboembolic events warrant particular caution.

Zitzmann et al. reported in the HEAT-Registry that long-acting intramuscular testosterone undecanoate led to a higher rate of hematocrit levels >50% compared to transdermal testosterone gel (69/304 vs. 25/498, *p* < 0.001) [18]. Advanced age, higher waist circumference, higher delta testosterone, and functional versus classical hypogonadism contributed to this effect [18].

Dobs et al. found that IM testosterone enanthate treatment was associated with significantly more abnormal hematocrit elevations (43.8% of patients) compared with transdermal testosterone (15.4% of patients) [17]. Vorkas et al. reported that in patients with HIV, intramuscular administration of testosterone demonstrated a stronger association with polycythemia than topical use, although no adverse cardiovascular or thrombotic events were observed [29].

Bassil et al. noted that injectable testosterone preparations are associated with higher erythrocytosis risk than topical preparations [32]. Vatti et al. mentioned that erythrocytosis (hematocrit >54–55%) is a known side effect of TRT, and patients on TRT may be at risk for pulmonary and deep venous thrombi [66].

### 5.5. Molecular Rationale for Preferring Transdermal Route

**Figure 1:**
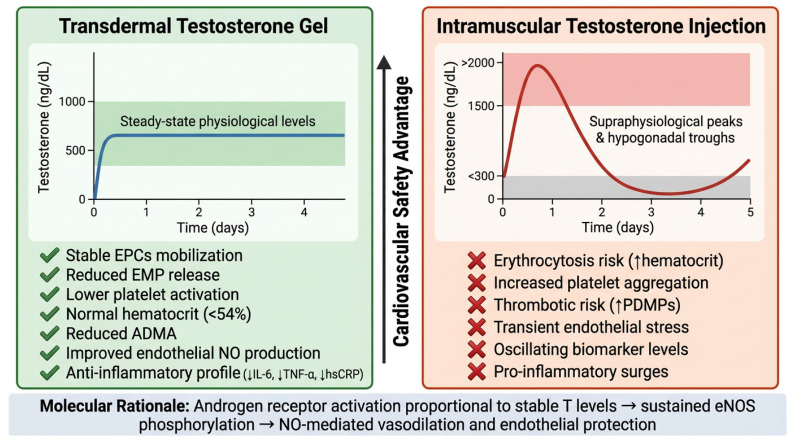
Transdermal Testosterone Gel vs. Intramuscular Injection: Pharmacokinetics and Cardiovascular Safety. Comparison of pharmacokinetic profiles and cardiovascular biomarker outcomes between transdermal testosterone gel and intramuscular testosterone injection. Left: Transdermal gel maintains steady-state physiological testosterone concentrations (300–1000 ng/dL green zone) with sustained androgen receptor (AR) activation, promoting eNOS phosphorylation, NO-mediated vasodilation, EPC mobilization, and an anti-inflammatory biomarker profile. Right: Intramuscular injection produces supraphysiological peaks (>1500 ng/dL) followed by sub-physiological troughs, associated with erythrocytosis risk (↑hematocrit), increased platelet aggregation, thrombotic risk (↑PDMPs), and pro-inflammatory surges. The central axis highlights the cardiovascular safety advantage of the transdermal route. Molecular rationale: Androgen receptor activation proportional to stable testosterone levels sustains eNOS phosphorylation, resulting in NO-mediated vasodilation and endothelial protection. Abbreviations: ADMA, asymmetric dimethylarginine; eNOS, endothelial nitric oxide synthase; NO, nitric oxide; PDMP, platelet-derived microparticle; ↓, decrease.

The molecular rationale for preferring transdermal testosterone formulations is based on maintaining physiological testosterone levels that optimize beneficial AR-mediated signaling while avoiding supraphysiological levels that promote oxidative stress, inflammation, and erythrocytosis [23,26]. Physiological testosterone levels enhance eNOS activity and NO production through the Src kinase-PI3K/Akt-eNOS pathway, inhibit NF-κB activation and inflammatory cytokine production, promote EPC mobilization and vascular repair, and improve endothelial function [19,81]. It is important to emphasize, however, that these mechanistic advantages do not yet constitute definitive evidence of superior clinical cardiovascular outcomes with transdermal versus intramuscular formulations. The mechanistic data provide a plausible biological basis for preferring the transdermal route, but large-scale comparative outcome trials are needed to confirm this in clinical practice (Figure 1).

Supraphysiological testosterone levels, in contrast, can induce mitochondrial ROS generation, activate the NLRP3 inflammasome, increase platelet reactivity, stimulate excessive erythropoiesis, and promote vascular dysfunction [26,36]. By maintaining steady-state physiological testosterone levels, transdermal formulations maximize cardiovascular benefits while minimizing potential risks [17].

Kalinchenko et al. stated that Androgel, a transdermal testosterone delivery system, is more effective than intramuscular and oral analogs, improves lipid spectrum, activates lipolysis to reduce visceral fat and insulin resistance, and has a vasodilating effect on the cardiovascular system and penile vessels [33]. The drug is described as safe and recommended for erectile dysfunction in patients with androgen deficiency and concurrent cardiovascular diseases [33].

## 6. Clinical Implications and Monitoring

The evidence reviewed has several important clinical implications for the management of hypogonadal men with cardiovascular risk factors or established cardiovascular disease [14]. First, circulating cardiovascular biomarkers, particularly EPCs and EMPs, provide valuable information about vascular health status and may help identify hypogonadal men at highest cardiovascular risk [8]. Reduced EPC numbers and elevated EMP levels suggest impaired vascular repair capacity and increased endothelial damage, warranting more aggressive cardiovascular risk factor modification [8].

Second, testosterone replacement therapy improves several cardiovascular biomarkers and endothelial function in hypogonadal men, supporting its potential cardiovascular benefits when appropriately prescribed [13,14]. However, TRT should be initiated only in men with confirmed hypogonadism (low testosterone levels on at least two morning measurements plus symptoms) and after careful cardiovascular risk assessment [80].

Third, current evidence suggests that transdermal testosterone formulations may offer a more favorable cardiovascular safety profile compared to intramuscular injections, primarily due to their more physiological pharmacokinetic profiles and lower rates of erythrocytosis [18,77]. While definitive head-to-head randomized trials with hard cardiovascular endpoints are still needed, available data on pharmacokinetic stability and lower erythrocytosis risk may support a cautious preference for transdermal formulations in men with cardiovascular risk factors or established cardiovascular disease; however, this should be regarded as a pharmacokinetic and hematological consideration rather than an evidence-based recommendation derived from outcome trials [15].

Fourth, regular monitoring during TRT is essential to ensure safety and efficacy [80]. Monitoring should include:

- Testosterone levels: Measured 3–6 months after initiation and then annually, with target levels in the mid-normal range (400–700 ng/dL) [80]
- Hematocrit: Measured at baseline, 3–6 months, and then annually; TRT should be interrupted if hematocrit exceeds 54% until levels normalize [80]
- Cardiovascular risk factors: Blood pressure, lipid profile, fasting glucose, and HbA1c should be monitored regularly [80]
- Cardiovascular symptoms: Patients should be counseled to report chest pain, dyspnea, leg swelling, or other cardiovascular symptoms promptly [80]

Fifth, certain patient populations require special consideration [80]. Men with recent cardiovascular events (myocardial infarction, stroke) should not initiate TRT until they are stable and at least 3–6 months post-event [80]. Men with severe heart failure, uncontrolled hypertension, or high baseline hematocrit (>50%) may not be suitable candidates for TRT [80].

Sixth, lifestyle modifications remain foundational for cardiovascular health in hypogonadal men [82]. Weight loss, regular exercise, smoking cessation, and dietary modifications can improve testosterone levels, reduce cardiovascular risk factors, and enhance the benefits of TRT [82].

## 7. Conclusions and Future Perspectives

Hypogonadism is associated with significant alterations in circulating cardiovascular biomarkers, including reduced endothelial progenitor cells, elevated endothelial microparticles, increased platelet reactivity, higher levels of endothelial activators and adhesion molecules, and increased inflammatory and oxidative stress markers [8]. These biomarker changes reflect impaired vascular repair capacity, increased endothelial damage, and a pro-thrombotic, pro-inflammatory state that contributes to increased cardiovascular risk in hypogonadal men [3] (Table 2).

Testosterone replacement therapy improves most of these cardiovascular biomarkers through multiple molecular mechanisms, including androgen receptor-dependent activation of the Src kinase-PI3K/Akt-eNOS signaling pathway, enhanced nitric oxide production, upregulation of VEGF and the CXCL12/CXCR4 axis promoting EPC mobilization, inhibition of NF-κB activation and inflammatory cytokine production, and improvement of antioxidant defenses [23,27]. These molecular effects translate into improved endothelial function, reduced inflammation, and potentially reduced cardiovascular risk [14].

However, the cardiovascular safety of TRT depends critically on the formulation used and the maintenance of physiological testosterone levels [17]. Current evidence suggests that transdermal testosterone formulations (gels, patches) provide steady-state physiological testosterone levels and may offer a more favorable cardiovascular safety profile compared to intramuscular injections, which produce supraphysiological peaks and are associated with higher rates of erythrocytosis and potentially increased thrombotic risk [18,77]. Definitive evidence demonstrating superior cardiovascular outcomes with transdermal versus intramuscular formulations from head-to-head randomized trials with hard endpoints remains limited. The molecular rationale for this difference lies in the dose-dependent effects of testosterone on oxidative stress, inflammation, and erythropoiesis, with supraphysiological levels promoting adverse effects [26] (Figure S1).

Future research directions should include:

Standardized biomarker assessment protocols: Development and validation of consensus protocols for EPC enumeration (harmonized surface marker panels) and EMP quantification (standardized pre-analytical and analytical procedures), to enable reliable cross-study comparisons and clinical implementation.

1. Prospective studies with cardiovascular endpoints: Large, long-term randomized controlled trials comparing transdermal and intramuscular testosterone formulations with hard cardiovascular endpoints (myocardial infarction, stroke, cardiovascular death) are needed [34].
2. Biomarker-guided TRT: Studies evaluating whether monitoring of EPCs, EMPs, and other cardiovascular biomarkers can guide TRT dosing and predict cardiovascular outcomes [8].
3. Personalized medicine approaches: Investigation of genetic polymorphisms (e.g., androgen receptor CAG repeat length, eNOS polymorphisms) that may influence individual responses to TRT and cardiovascular risk [22].
4. Novel testosterone formulations: Development and evaluation of testosterone formulations that more closely mimic physiological circadian variations and minimize adverse effects [83].
5. Combination therapies: Studies evaluating whether combining TRT with other interventions (e.g., PDE-5 inhibitors, statins, antioxidants) can enhance cardiovascular benefits [27].
6. Mechanistic studies: Further elucidation of the molecular mechanisms by which testosterone influences EPC biology, endothelial function, platelet activation, and vascular inflammation [84].

Comparative effectiveness studies: Long-term randomized controlled trials directly comparing transdermal and intramuscular testosterone formulations with hard cardiovascular endpoints (myocardial infarction, stroke, cardiovascular death) are needed to establish whether the pharmacokinetic advantages of transdermal formulations translate into superior clinical outcomes. Identification of patient subgroups most likely to benefit from TRT, based on genetic polymorphisms, baseline biomarker profiles, and cardiovascular risk stratification, represents another key research priority.

In conclusion, circulating cardiovascular biomarkers provide valuable insights into the vascular health of hypogonadal men and the cardiovascular effects of testosterone replacement therapy [8]. Understanding the molecular mechanisms underlying these biomarker changes is essential for optimizing TRT and minimizing cardiovascular risk [19]. Based on currently available evidence, transdermal testosterone formulations may be preferred over intramuscular injections due to their more physiological pharmacokinetic profiles and lower erythrocytosis risk; however, this preference should be qualified by acknowledging that robust head-to-head randomized trials with hard cardiovascular endpoints are still lacking [77]. With appropriate patient selection, formulation choice, and monitoring, TRT can improve cardiovascular biomarkers and potentially reduce cardiovascular risk in hypogonadal men [14]. It must be explicitly acknowledged, however, that improvements in circulating biomarkers observed with TRT represent changes in surrogate endpoints and should not be interpreted as direct evidence of improved long-term cardiovascular outcomes unless corroborated by clinical endpoint data from adequately powered randomized controlled trials.

**Table 1:** Summary of Principal Clinical Studies on Testosterone Replacement Therapy and Circulating Cardiovascular Biomarkers in Hypogonadal Men.

First Author & Year	Study Design	Sample Size (n)	TRT Type and Dose	Duration of Treatment	Biomarkers Measured	Key Findings on Biomarker Changes
Foresta 2006 [52]	Prospective	10 hypogonadal men and 25 controls (*n* = 35) [52]	Transdermal testosterone gel, 50 mg/day [52]	6 months [52]	Circulating progenitor cells (PCs) and endothelial progenitor cells (EPCs) by flow cytometry [52]	Hypogonadal men had reduced EPC/PC counts versus controls at baseline; 6 months of T gel increased circulating PCs/EPCs toward control levels [52].
Liao 2013 [13]	Prospective observational	29 men receiving TRT (46 recruited; 29 treated) [13]	Transdermal gel (AndroGel 1%), 5 g/day [13]	12 months [13]	Circulating EPCs (flow cytometry; EPCs per 100,000 monocytes) [13]	EPC counts rose from baseline (9.5 ± 6.2) to 3, 6 and 12 months (16.6 ± 11.1; 20.3 ± 15.3; 27.2 ± 15.5), statistically significant increases over time [13].
La Vignera 2011 [8]	Prospective comparative	50 men with ED and LOH (30 treated, 20 untreated) [8]	Transdermal androgen (Tostrex) regimen (topical) [8]	6 months [8]	EPCs (CD45neg/CD34pos/CD144pos) and EMPs (CD45neg/CD34neg/CD144pos) by flow cytometry [8]	After 6 months, treated group had higher EPCs and lower EMPs relative to untreated LOH patients, with better vascular/erectile parameters in treated men [8].
Di Mambro 2010 [51]	Cross-sectional with follow-up	68 Klinefelter men and 46 controls; 22 hypogonadal KS re-evaluated after TRT [51]	Androgen therapy (various formulations used clinically) [51]	6 months follow-up in subgroup [51]	Circulating EPC number	KS patients had lower EPC counts than controls; in 22 hypogonadal KS men re-evaluated after 6 months of androgen therapy there was no significant change in EPC number [51].
Di Minno 2015 [11]	Cross-sectional case–control	23 Klinefelter men on TRT and 46 controls (*n* = 69) [11]	Ongoing testosterone replacement therapy in KS patients (formulations per clinical care) [11]	Cross-sectional (on-treatment) [11]	Platelet reactivity (light transmission aggregometry) and urinary platelet markers (8-iso-PGF2α, 11-dehydro-TXB2) [11]	KS men on TRT showed increased platelet reactivity and higher urinary platelet activation markers versus controls, suggesting raised platelet activation in this cohort [11].
Sader 2003 [84]	Prospective within-subject	9 hypogonadal men (each as own control) [84]	Intramuscular depot testosterone, 800 mg every 6 months [84]	Single dosing with trough vs. peak comparisons (2–4 weeks post-dose) [84]	Vascular reactivity: flow-mediated dilatation (FMD) and nitroglycerin response (ultrasound)	Physiological replacement produced decreased endothelium-dependent dilation (reduced FMD) at peak vs. trough testosterone levels in these men [84].
Francomano 2016 [22]	Pilot acute study	Small pilot cohort (severe hypogonadism; n not specified in abstract) [22]	Single/short-term administration of testosterone gel (acute dosing) [22]	Acute (hours) response assessed	Acute endothelial response measures and relation to androgen receptor polymorphism	Reported acute vascular effects of testosterone gel with variability related to androgen receptor polymorphism; study assessed immediate endothelial responses after gel administration [22].
Shoskes 2016 [57]	Prospective clinical cohort	23 symptomatic hypogonadal men (20 compliant retested) [75]	Testosterone therapy (formulation individualized; dose titrated) [75]	Retesting between 3 and 6 months after initiation [75]	Endothelial function by reactive hyperemia index (RHI) and augmentation index (AI)	Baseline endothelial dysfunction common; after TRT endothelial function either remained unchanged or improved (mean RHI increased in compliant patients) with no cardiac events during follow-up [75].
Zitzmann HEAT-Registry 2022 [18]	Prospective registry, two-arm	802 hypogonadal men (498 T gel, 304 IM testosterone undecanoate) [18]	Transdermal T gel vs. intramuscular long-acting testosterone undecanoate (TU) per routine dosing [18]	Minimum follow-up 26 weeks (median follow-up weeks 26–30) [18]	Hematologic parameters (hematocrit), serum total testosterone	Both formulations raised testosterone; intramuscular TU was associated with higher rates of hematocrit >50% (69/304 vs. 25/498 with gel), with older age, waist circumference and larger T increases predicting hematocrit rise [18].
Gianatti 2016 [83]	Randomized double-blind placebo-controlled trial	88 men with T2D and low T (45 TU vs. 43 placebo) [83]	Intramuscular testosterone undecanoate (TU) vs. placebo (standard regimen) [83]	40 weeks (≈9 months) [83]	Cardiac biomarkers: NT-proBNP and high-sensitivity cardiac troponin T (hs-cTnT)	Testosterone reduced NT-proBNP (mean adjusted difference −17.9 ng/L) and did not change hs-cTnT versus placebo over 40 weeks [83].
Mohler (Testosterone Trials) 2018 [29]	Double-blind randomized placebo-controlled trial	788 men ≥65 years with low T (part of Testosterone Trials) [29]	Transdermal testosterone gel, dose adjusted to restore young-adult range vs. placebo gel [29]	12 months [29]	Lipids, insulin, HOMA-IR, d-dimer, CRP, IL-6, troponin, and other metabolic/inflammatory markers	Compared with placebo, testosterone produced small decreases in total cholesterol and HDL and slight reductions in fasting insulin and HOMA-IR, but no significant changes in triglycerides, d-dimer, CRP, IL-6, or troponin over 12 months [29].
Shaikh 2020 (Testosterone Trials substudy) [29]	Observational substudy of randomized trial	Subset of Testosterone Trials participants (older men)—sample per substudy cohort (n in paper) [29]	Transdermal testosterone gel vs. placebo as in main Trials [29]	12 months (Trial duration) [29]	Multiple cardiovascular biomarkers and coronary noncalcified plaque (NCP) progression measurements	Found testosterone treatment was associated with greater progression of noncalcified coronary plaque; biomarker analyses explored associations with plaque progression (paper details biomarker-plaque links) [29].
TRAVERSE 2023 (Nguyen/et al.) [34]	Multicenter randomized double-blind placebo-controlled non-inferiority trial	5246 men randomized (testosterone vs. placebo) [34]	Daily transdermal 1.62% testosterone gel, dose adjusted to achieve 350–750 ng/dL target vs. placebo gel [34]	Mean treatment 21.7 ± 14.1 months (mean follow-up 33.0 months) [34]	Primary outcomes were major adverse cardiovascular events; routine labs and adverse event surveillance reported	TRT was noninferior to placebo for the composite of death from cardiovascular causes, nonfatal MI, or nonfatal stroke; increased incidence of atrial fibrillation, acute kidney injury and pulmonary embolism observed in testosterone group; the trial was focused on clinical events rather than detailed serial biomarker panels [34].
Olopade 2022 [14]	Systematic review (clinical studies)	23 publications included, pooled participants n≈102,139 across studies [14]	Various TRT formulations and doses across included studies [14]	Durations varied across included studies (short to long term) [14]	Variable: clinical cardiovascular outcomes and biomarkers examined across included studies	Review concluded long-term TRT in LOH/FH appears to offer cardiovascular benefits in selected patients but advised caution in men with pre-existing CVD; findings were heterogeneous across studies and endpoints [14].
Quang 2018 [15]	Systematic review/retrospective review	25 studies included (12 RCTs and 13 non-RCTs) with total n reported across reviews [15]	Varied TRT types and doses across studies	Varied across included studies [15]	Cardiovascular outcomes and surrogate biomarkers in T2D and metabolic syndrome cohorts	Review suggested possible protective effects of TRT on mortality and major adverse CV events in hypogonadal men with T2D/MS in several included studies but urged cautious interpretation due to heterogeneity and limited RCT data [15].

**Table 2:** Summary of Circulating Cardiovascular Biomarkers in Hypogonadism and Effects of Testosterone Replacement Therapy (TRT).

Biomarker	Direction in Hypogonadism	Effect of TRT	Proposed Molecular Mechanism	Potential Clinical Significance
EPCs (Endothelial Progenitor Cells)	↓ Reduced circulating EPC numbers	↑ Increased after TRT (most studies)	CXCL12/CXCR4 axis upregulation; eNOS/NO enhancement; VEGF upregulation promoting BM mobilization	Impaired vascular repair; increased CV risk; EPC increase may reflect improved endothelial regeneration
EMPs (Endothelial Microparticles)	↑ Elevated (CD45neg/CD144+/annexin V+)	↓ Expected reduction (limited data)	TRT improves endothelial function → reduced endothelial activation/apoptosis	Reflect endothelial damage; elevated EMPs indicate increased thrombotic and inflammatory risk
Platelet activation markers (P-selectin, PDMPs, TXA2)	↑ Increased platelet reactivity	Controversial; may ↑ at supraphysiological doses; neutral at physiological TRT	Testosterone modulates TXA2 receptor density, P2Y12 expression; NO reduces platelet aggregation	Increased platelet activation → thrombotic risk; distinction between physiological and supraphysiological exposure is critical
ICAM-1, VCAM-1, E-selectin	↑ Elevated adhesion molecules	↓ Reduced with TRT	AR-mediated suppression of NF-κB; reduced endothelial activation	Promote leukocyte adhesion and atherosclerotic plaque formation
vWF (von Willebrand Factor)	↑ Elevated	↓ Reduced with TRT	Reduced endothelial activation; NO-mediated suppression of vWF release	Prothrombotic; elevated vWF promotes platelet adhesion and coagulation
ET-1 (Endothelin-1)	↑ Elevated	↓ Reduced with TRT	Testosterone inhibits ET-1 synthesis; promotes NO-mediated vasodilation	Vasoconstriction; promotes endothelial dysfunction and hypertension
ADMA (Asymmetric Dimethylarginine)	↑ Elevated	↓ Reduced with TRT	Testosterone reduces ADMA via enhanced DDAH activity; restores eNOS function	Endogenous eNOS inhibitor; elevated ADMA → reduced NO → endothelial dysfunction
hsCRP	↑ Elevated	↓ Reduced in some studies; no change in Testosterone Trials	NF-κB suppression; reduced IL-6-driven hepatic CRP synthesis	Systemic inflammation marker; heterogeneous TRT response; biomarker improvement ≠ CV event reduction
IL-6, TNF-α	↑ Elevated	↓ Reduced in some studies; inconsistent	AR-mediated NF-κB inhibition; reduced macrophage activation	Pro-inflammatory cytokines; contribute to endothelial dysfunction and atherosclerosis
Hematocrit/Erythrocytosis	Normal or ↓ (in untreated hypogonadism)	↑ Risk with TRT, especially IM formulations	↑ EPO production; ↓ hepcidin; direct BM erythroid stimulation	Increased blood viscosity → thrombotic risk; formulation-dependent (IM > transdermal)
SOD/Antioxidant enzymes	↓ Reduced activity	↑ Restored with physiological TRT	Testosterone upregulates SOD and GSH-Px expression; reduces mitochondrial ROS	Impaired antioxidant defense → oxidative stress → vascular damage

Abbreviations: ADMA, asymmetric dimethylarginine; EMP, endothelial microparticle; EPC, endothelial progenitor cell; hsCRP, high-sensitivity C-reactive protein; IM, intramuscular; TRT, testosterone replacement therapy; ↑, increase; ↓, decrease.

## Data Availability

No new data were created or analyzed in this study. Data sharing is not applicable to this article.

## Supplementary Materials

The following supporting information can be downloaded at: https://www.mdpi.com/article/10.3390/ijms27136035/s1.
