# Supplementary material for: Circulating Markers of Cardiovascular Health in Hypogonadism Before and After Testosterone Therapy: Molecular Aspects and Formulation Comparison
Source: Int J Mol Sci. 2026 Jul 5;27(13):6035. doi: 10.3390/ijms27136035 (PMC13362008; doi:10.3390/ijms27136035)
Supplement: Supplementary file 1 [file ijms-27-06035-s001.zip › ijms-4396578-supplementary.pdf]

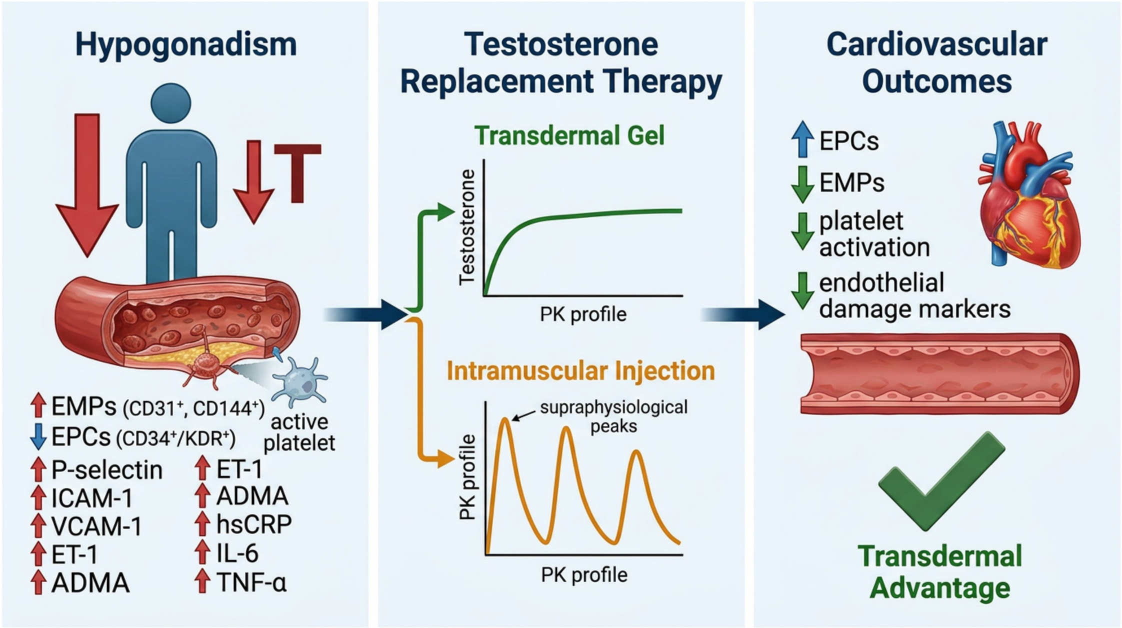

Supplementary Figure S1. Graphical Abstract. Schematic representation of the pathophysiological cascade linking hypogonadism to alterations in circulating cardiovascular biomarkers and the effects of testosterone replacement therapy (TRT). Left panel: Hypogonadal state (↓T) is associated with elevated endothelial microparticles (EMPs; CD31+, CD144+), reduced endothelial progenitor cells (EPCs; CD34+/KDR+), increased platelet activation (↑P-selectin), and elevated endothelial activators (↑ICAM-1, ↑VCAM-1, ↑ET-1, ↑ADMA, ↑hsCRP, ↑IL-6, ↑TNF-α). Central panel: TRT via transdermal gel produces steady-state physiological testosterone levels, whereas intramuscular (IM) injection generates supraphysiological peaks followed by hypogonadal troughs. Right panel: TRT—particularly via the transdermal route — restores a favorable biomarker profile (↑EPCs, ↓EMPs, ↓platelet activation, ↓endothelial damage markers), conferring a cardiovascular safety advantage. Abbreviations: ADMA, asymmetric dimethylarginine; EMP, endothelial microparticle; EPC, endothelial progenitor cell; ET-1, endothelin-1; hsCRP, high-sensitivity C-reactive protein; ICAM-1, intercellular adhesion molecule-1; IL-6, interleukin-6; T, testosterone; TNF-α, tumor necrosis factor-alpha; VCAM-1, vascular cell adhesion molecule-1.
